# Bioinformatics Analysis, Expression Profiling, and Functional Characterization of Heat Shock Proteins in *Wolfi-poria cocos*

**DOI:** 10.3390/bioengineering10030390

**Published:** 2023-03-22

**Authors:** Xin Hu, Xue Tang, Yumei Zhou, Bilal ahmad, Deli Zhang, Yue Zeng, Jingyi Wei, Liling Deng, Shijiang Chen, Yu Pan

**Affiliations:** 1 College of Horticulture and Landscape Architecture, Southwest University, Chongqing 400715, China; 2 Key Laboratory of Horticulture Science for Southern Mountainous Regions, Chongqing 400715, China; 3 Agricultural Genomics Institute at Shenzhen, Chinese Academy of Agricultural Sciences, Shenzhen 518120, China; 4 Chongqing Academy of Chinese Materia Medica, Chongqing 400062, China; 5 Chongqing Academy of Agricultural Sciences, Chongqing 401329, China; 6 Chongqing Institute of Biotechnology Co., Ltd., Chongqing 401121, China

**Keywords:** *Wolfiporia cocos*, high temperature stress, heat shock protein, prokaryotic expression

## Abstract

Heat shock proteins (HSPs) play critical roles in regulating different mechanisms under high-temperature conditions. HSPs have been identified and well-studied in different plants. However, there is a lack of information about their genomic organization and roles in medicinal plants and fungi, especially in *Wolfi-poria cocos (W. cocos).* We identified sixteen heat shock proteins (HSPs) in *W. cocos* and analyzed in terms of phylogenetic analysis, gene structure, motif distribution patterns, physiochemical properties, and expression comparison in different strains. Based on phylogenetic analysis, HSPs were divided into five subgroups (WcHSP100, WcHSP90, WcHSP70, WcHSP60, and WcsHSP). Subgroups WcHSP100s, WcHSP90s, WcHSP70s, WcHSP60, and WcsHSPs were further divided into 3, 2, 3, 1, and 6 subfamilies, respectively. Moreover, the expression profiling of all HSP genes in five strains of *W. cocos* under different temperature extremes revealed that expression of most HSPs were induced by high temperature. However, every subfamily showed different expression suggesting distinctive role in heat stress tolerance. WcHSP70-4, WcHSP90-1, and WcHSP100-1 showed the highest response to high temperature stress. Heterologous expression of WcHSP70-4, WcHSP90-1, and WcHSP100-1 genes in *Escherichia coli* enhanced survival rate of *E. coli* during heat stress. These findings suggest the role of *W. cocos* heat shock genes in the high temperature stress tolerance.

## 1. Introduction

Heat shock proteins (HSPs) are newly synthesized and increased by organisms or cells within a certain period of time under high-temperature stress [1]. According to the size and structure of proteins, HSP family members are divided into five groups, namely HSP100, HSP90, HSP70, HSP60, and sHSP, involved in various aspects of cellular function. For example, most of the HSP100 proteins are ATP-dependent proteases with ATPase activity and molecular chaperone activity, which interact with other proteins and cooperate with other molecular chaperones to repair denaturing proteins and prevent faulty proteins from polymerizing [2,3]. The main function of HSP90 is the final maturation of proteins and the structural assembly of complex supramolecular proteins, and it also interacts with some kinases and transcription factors [4]. HSP70 mainly promotes protein degradation by binding to proteins to be degraded and localizing them to the proteasome and lysosome [5,6]. HSP60 consists of two subfamilies: one found in the cytoplasm of eukaryotes and archaea [7], and the other found in chloroplasts, mitochondria, and bacteria [8]. The primary function of HSP60 is the provision of assistance in prop folding of other proteins and prevention of aggregation and accumulation of misfolded conformation of proteins [9,10]. sHSPs protect organisms from abiotic stress damage by preventing irreversible protein aggregation and scavenging cellular reactive oxygen species. However, HSPs are synthesized in large quantities when an organism is subjected to high-temperature stress and act as molecular chaperones [11]. By binding to the reactive active region of proteins, they prevent misfolding or random aggregation of proteins by binding with active regions of proteins and lead the proteins to form an appropriate conformation. In this way, protein denaturation can be prevented and adverse effects caused by high-temperature stress on the growth and development of organisms can be alleviated, protein homeostasis can be maintained and cells can be protected from high-temperature damage [12].

In general, temperature plays an important role in the growth and development of fungi, including fungal growth, spore germination and reproduction [13]. For example, when the mycelium of *Pleurotus eryngi* is subjected to high-temperature stress, its growth rate decreases and the mycelium cells become short and thick [14]. Although *Ganoderma lucidum* is a large fungus that requires high temperature and good air for normal growth, when the temperature exceeds 35 °C, the growth of mycelium is inhibited and leads to problems such as thin cap and poor quality [15]. Several studies showed that *G. clavigera* is normally grown at lower temperatures, but *O. montium* grows at warmer temperatures [16,17,18]. In addition, high temperature affects both morphological structure and physiological activities of fungus, such as an increase in protein denaturation and reactive oxygen species (ROS) production with cell metabolism and related gene expression [19]. *W. cocos*, also called tuckahoe, grows on the roots of pines and is distributed in East Asia, India, and some American states [20]. The sclerotium of *W. cocos* is a precious source of traditional Chinese medicine with the roles in strengthening the spleen and heart soothing [21]. However, the mechanism of high temperature involved in affecting the growth rate and sporulation is still unknown in *W. cocos*. Furthermore, the production of HSPs is a kind of adaptation of life to the environment. In this study, therefore, we identified and characterized the HSP family genes from *W. cocos*, and determined the expression patterns of these genes under high temperature stress. Our results lay a foundation to understand the molecular mechanism of *W. cocos* HSPs in response to high temperature, and improve the production practices of *W. cocos* industry.

## 2. Materials and Methods

### 2.1. Experimental Materials and Heat Treatment 

Five *W. cocos* strains, including 28 (ACCC 51641), 86 (ACCC 51642), 5.78 (ACCC 50864), 5.908 (CGMCC 5.908), and S10 (ACCC 51976), were obtained from the Chongqing Academy of Chinese Materia Medica, Chongqing, China. They were grown on Potato Dextrose Agar (PDA) medium at 28 °C and kept in the refrigerator at 4 °C for subsequent experiments. *E. coli* strains DH5*α* and BL21 (DE3) were provided by the Horticultural Plant Development and Stress Biology Innovation Team Laboratory, College of Horticulture and Landscaping, Southwest University.

All the five varieties of *W. cocos* were used in the heat treatment, and we inoculated the mycelian on PDA medium with a perforator. When the mycelian grew to 6 cm under the optimum temperature, they were placed in the incubator at different temperatures. The mycelial growth was measured by a vernier caliber. At least three biological repetitions were performed for each treatment. The method of multiple comparison of Least Significant Difference (LSD) was used to analyze the differences.

The same method was used in the high temperature treatment of mycelium, and the samples at 0 h, 1 h, 6 h, 12 h, 24 h and 48 h were collected; those collected at 0 h were used as the control. At least three biological repetitions were performed for each treatment and all samples were collected and frozen in liquid nitrogen immediately for RNA extraction.

### 2.2. W. cocos Sequence Analysis and Phylogenetic Tree Construction

The mycelia of *W. cocos* ‘28’ were sent to Novogene Co., Ltd Beijing, China. for transcriptome sequencing, the coding sequence (CDS), and amino acid sequence of the corresponding heat shock protein-related genes were retrieved (Table S1 and Table S2). Further, the Basic Local Alignment Search Tool (Blast) was performed and nucleotides with high homology and similar species were selected for further studies. The National Centre for Biotechnology Information (NCBI) conserved domain database (https://www.ncbi.nlm.nih.gov/Structure/cdd/cdd.shtml (accessed on 11 January 2022) was used to check the integrity and completeness of heat shock protein domain in the selected sequences. Phylogenetic trees were constructed and verified by way of the Neighbor-Joining (NJ) method using MEGA V6.0 with 1000 repeated guidance tests. The conserved domains of heat shock transcription factors and heat shock proteins of *W. cocos.* were analyzed by MEME (https://meme-suite.org/meme/tools/meme (accessed on 15 January 2022).

### 2.3. Physiochemical Properties of Proteins and in Silico Protein-Protein Analysis

ExPASy ProtParam (https://web.expasy.org/protparam/ (accessed on 15 March 2022) and PredictProtein (https://www.predictprotein.org/ (accessed on 15 March 2022) were used to determine the relevant physical and chemical properties of proteins, including molecular weight (MW), isoelectric point (pI), and the subcellular localization analysis. For prediction of protein–protein, online program STRING (https://string-db.org/ (accessed on 25 March 2022) was used.

### 2.4. RNA Extraction and cDNA Synthesis

Total Ribonucleic Acid (RNA) was extracted from five kinds of *W. cocos* hyphae using the ominplant RNA Kit (DNaseI) Kit (Cwbio). The concentration and quality of RNA were verified by K5800-ultrafine spectrophotometer and 1.5% agarose gel electrophoresis, respectively. All obtained RNAs were retroscribed using DNaseI, RNASe-Free kits, and HiFiScript genomic deoxyribonucleic acid (gDNA) Removal complementary deoxyribonucleic acid (cDNA) Synthesis Kit (Cwbio) reverse transcription Kit. cDNA was stored in a −20 °C refrigerator for subsequent experiments. 

### 2.5. Expression Analysis by Quantitative Real-Time Polymerase Chain Reaction (qRT-PCR)

Gene-specific primers were designed using Primer Premier 7.0 (Table S3). The Qtower3G fluorescence quantitative Polymerase Chain Reaction instrument was used for qRT-PCR analysis. PCR conditions were the following: 95 °C for 10 min, 95 °C for 15 s, followed by 40 cycles of 59–63 °C (depending on the specific gene) for 30 s, and melting curve analysis were performed over a temperature range of 65–95 °C in 0.5 °C increments. The reaction mixture was 5 μL of 2 × UltraSYBR Mixture (Low ROX), 0.5 μL of each primer (10 μM), 2 μL of cDNA, and 2 μL of ddH_2_O. All cDNA samples were set with three technical replicates and a negative No Template Control (NTC) was set at the same time to analyze the relative expression levels of genes under the optimal annealing temperature. The amplification efficiency of each gene was within the 0.95–1.05 range, and R^2^ was greater than 0.99. GADPH and ACT were used as an internal control and the relative expressions were calculated by the 2^−ΔΔCt^ method [22]. All data are means ± SD of three technical replicates; the 0 h sample was taken as control, two-way ANOVA was employed to analyze the data, and significant differences are represented by asterisks: * *p* < 0.05; ** *p* < 0.01.

### 2.6. Cloning of Heat Shock Protein Target Gene

According to the qRT-PCR results, the differentially expressed genes were selected for full-length specific amplification using Phanta^®^ Max super-fidelity DNA polymerase. The amplification conditions were as follows: 95 °C for 3 min, then 35 cycles of 95 °C for 15 s, 59 °C for 15 s, 72 °C for 2 min, and finally 72 °C for 5 min. The amplified products were verified and purified by 1.5% agarose gel and Universal DNA Purification Kit (Tiangen, Beijing, China), respectively. Specific primers were used for amplification (Table S2). The product was cloned into the pMD19-T vector (Southwest University, Chongqing, China). The gene was transformed into *E. coli* DH5*α* competent cells (Southwest University, Chongqing, China), and the positive clone plaque was identified by Taq enzyme using universal primer M13F along with gene-specific primers before and after the gene sequencing. Plasmid extraction was performed using the correctly sequenced bacteria through the FastPure Plasmid Mini Kit. Plasmid Extraction Kit was used to determine the Plasmid concentration.

### 2.7. Construction of Recombinant Plasmid Prokaryotic Expression Vector

The selected fragments of the target gene and the vector information of pET-32a (+) were analyzed and double digested with *Not*I and *Kpn*I restriction sites. Primers for the target genes containing the restriction sites were designed using Primer Premier 7.0 (Table S2). The purified plasmid was used as a template and the Phanta^®^ Max Super-Fidelity DNA was used to specifically amplify the full length of the restriction site. The amplified PCR products were detected by gel electrophoresis. The Universal DNA Purification Kit (Tiangen, Beijing, China) was used for DNA extraction and purification according to the manufacturer’s guidelines. The ligation product was transferred into *E. coli* DH5*α*. Taq enzyme was used to identify the positive clone plaque by using the general primer S-tag and the back primer of the target gene and the general primer T7-TER and the forward primer of the target gene. Finally, the constructed recombinant vector plasmid and pET-32a (+) plasmid were introduced into the *E. coli* BL21.

### 2.8. Prokaryotic Expression and Heat Resistance

*E. coli* BL21 containing pET-32a (+) expression vector was inoculated in Luria–Bertani (LB) medium containing ampicillin (Amp) resistance at 1% volume fraction, expanded to optical density 600 (OD_600_), 0.6–0.8 at 37 °C, induced by 1 mM Isopropyl-beta-D-thiogalactopyranoside (IPTG) for 3.5 h, and then adjusted to OD_600_, 0.6–0.8 again. The difference of each OD_600_ was kept within 0.002. After induced expression, 200 μL of the bacterial solution was transferred to a centrifuge tube and was heated in a water bath at 50 °C for 0 min, 30 min, 60 min, 90 min, 120 min, and 150 min, respectively. A total of 2 μL of samples was extracted into LB medium plate and was cultured at 37 °C for 12–16 h, with 3 replicates per gradient.

## 3. Results

### 3.1. The Heat-Resistant Analysis of Different W. cocos Strains

**Figure 1:**
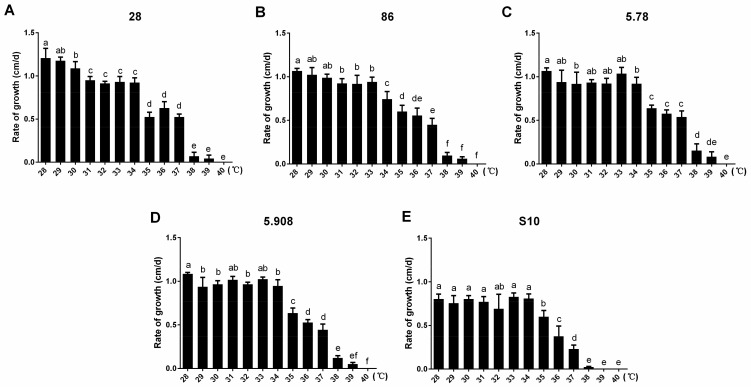
The growth rate of strains under different temperature treatments. (**A**–**E**) indicates the growth rate of different strains ‘28’, ‘86’, ‘5.78’, ‘5.908’, and ‘S10’ of *W. cocos* under temperature treatment. All data are means ± SD of at least three technical replicates, and use the method of multiple comparison of LSD to analysis the differences, significant difference is represented by different letters a–f, ‘cm/d’ means centimeter per day.

In order to analyze the resistance of different *W. cocos* strains to the high temperature, experiments of different temperature treatments were carried out in five strains of *W. cocos.* By measuring the speed of the mycelial growth shown in Figure 1, we determined that the ‘28’ strain was the fastest to grow and the ‘S10’ was the one to do so the most slowly out of the five strains. However, the growth of strains mycelium could be inhibited by the high temperature (Figure 1). Speed of growth, the density and color of mycelia (Figure S1) showed that the majority of strains were influenced at 35 °C and severely inhibited at 38 °C, but different strains also had some differences. The ‘28’ and ‘5.78’ strains showed steady growth at 30–34 °C; despite the high temperature stress, the mycelium could maintain a certain growth speed at 35–37 °C. Endurance of ‘S10’ and ‘86’ under high temperature is relatively worse than that of the other three strains. As the temperature increased, the speed of growth decreased, then mycelium began browning at 38 °C. Strain ‘10’ already stopped growing at 39 °C. The heat resistance of five *W. cocos* strains was as follows: ‘28’ = ‘5.78’ > ‘5.908’ > ‘86’ > ‘S10’.

### 3.2. Identification and Characterization of HSP Family Genes in W. cocos 

In this study, a total of 16 WcHSPs were identified in *W. cocos* based on HMM profile and BLASTP analysis (Table S1). Among them, three, two, four, one, and six proteins belonged to HSP100, HSP90, HSP70, HSP60, and sHSP families, respectively. These *HSP* genes from *W. cocos* were defined as WcHSPs and listed in Table 1. As shown in Table 1, the amino acid residues, including WcHSP100s, WcHSP90s, and WcHSP70s, are varied from 128 to 287, 904 to 1043, 695 to 800, and 612 to 780, respectively, while the amino acid residues of WcHSP60s member was 456. Further, the isoelectric point ranged from 4.75 (WcHSP90-1) to 9.03 (WcsHSP-3). The fat solubility index of all members was more than 70 and WcsHSP-4 had the highest (110) fat solubility index. Members of WcHSP100 and WcsHSP were predicted in the nucleus, while WcHSP70 proteins were mainly distributed in the cytoplasm.

### 3.3. Phylogenetic Analysis of Heat Shock Protein Family Genes in W. cocos

**Figure 2:**
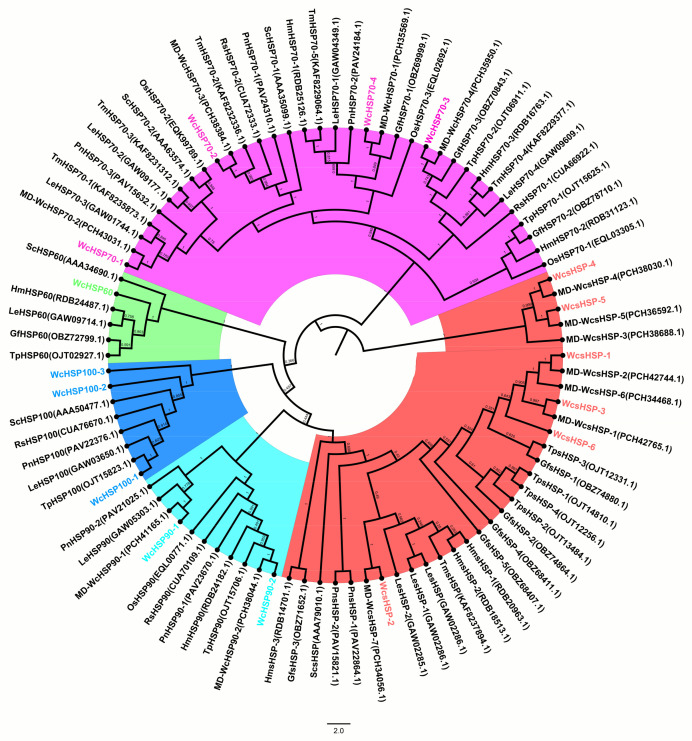
Phylogenetic analysis of heat shock proteins from different species. Different colors represent different subfamilies; red color denotes WcsHSP subgroup; green color denotes WcHSP60 subgroup; purple color denotes WcHSP70 subgroup; turquoise color denotes WcHSP90 subgroup and blue color denotes WcHSP100 subgroup. The numbers near the branches denote bootstrap values.

To reveal the evolutionary relationships of WcHSP members, the NJ phylogenetic tree was constructed using WcHSP proteins from eleven different species, including *W. cocos*, *Trametes pu-bescens*, *Grifola frondosa*, *Rhizoctonia Solani*, *Lentinula edodes*, *Saccharomyces cerevisiae*, *Hypsizygus marmoreus*, *Tricholoma Matsutake*, *Pyrrhoderma noxium*, *Ganoderma boninense* and *Ophiocordyceps sinensis*, respectively. As shown in Figure 2, the 16 WcHSPs were classified into five groups, including three HSP100s, two HSP90s, four HSP70s, one HSP60 and six sHSPs, respectively. Based on phylogenetic analysis, the HSP90 and HSP100 family genes showed high similarity with each other. However, the HSP60 family genes were closely related with some sHSP family genes. In addition, these identified HSP family genes from *W. cocos* ‘28’ were clustered into the same groups with those homologous genes from MD-104 SS10 (United States), indicating that the genetic link between the cultivars and confirming that the heat shock protein sequences were conserved in different *W. cocos*.

### 3.4. Conserved Motif Analysis of Heat Shock Protein in W. cocos

**Figure 3:**
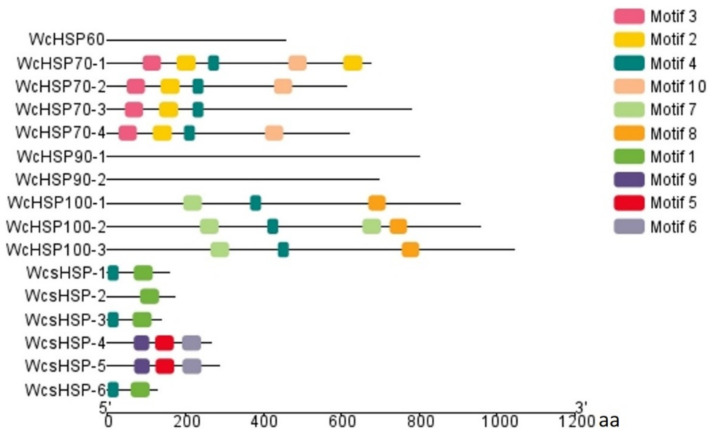
Analysis of conserved motifs of heat shock proteins. Different colors denote different motifs and length of box denotes motif length. ‘aa’ means amino acid.

In addition, we determined that most of the genes showed similar motif patterns within the same subgroup but different among the families (Figure 3). For example, no conserved motif was identified in WcHSP90 and WcHSP60 family genes (Figure 3), hence the functional of these family genes are quite different from the other subgroups. All members of WcHSP70 have almost similar motif patterns with few exceptions, and three motifs (Motif 2, 3, and 4) were present in all four members. However, Motif 10 was present in all members except WcsHSP-3. In addition, three motifs (Motif 4, 7, and 8) were detected in all members of WcHSP100, while WcHSP100-2 contained one extra Motif 7. Moreover, WcsHSP-1, WcsHSP-2, WcsHSP-3, and WcsHSP-6 showed more similarity with each other compared to other members of WcsHSP subfamily. However, WcsHSP-4 and WcsHSP-5 were also very similar to each other. These conserved motifs may hold the key to understanding the way different heat shock proteins contribute to different biological functions.

### 3.5. Expression Patterns of Heat Shock Proteins at Different Temperatures in W. cocos

In order to analyze the function of HSPs in the high temperature and the response to high temperature stress, we tested the expression of HSPs in five strains following heat treatments for different timeframes using qRT-PCR.

**Figure 4:**
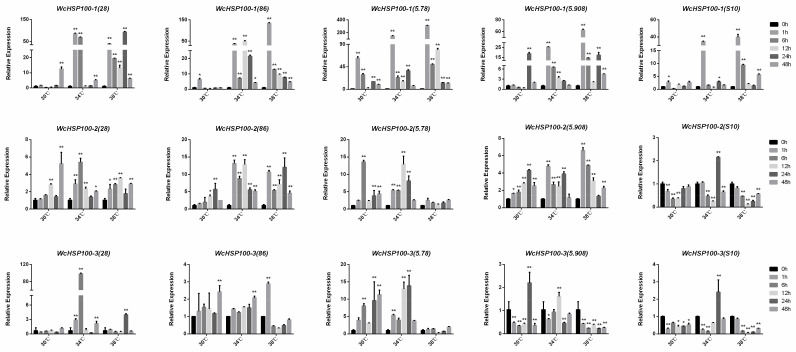
Analysis of expression patterns of *WcHSP100* gene under different temperature treatments. The different strains ‘28’, ‘86’, ‘5.78’, ‘5.908’, and ‘S10’ of *W. cocos* are shown. All data are means ± SD of three technical replicates; the 0 h sample is taken as control, two-way ANOVA was employed to analyze the data, and significant differences are represented by asterisks: * *p* < 0.05; ** *p* < 0.01.

The results of expression of *HSP100* showed that the level of *WcHSP100-1* expression was higher than that of *WcHSP100-2* and *WcHSP100-3*, especially at 1 h, and had a transient expression effect (Figure 4). *WcHSP100-1* expression was significantly induced after high temperature treatments and higher in all *W. cocos* strains after 1 h of treatment at varied temperatures. However, *WcHSP100-1* showed the highest expression in *W. cocos* ‘5.78’ and lowest in ‘S10’ after 1 h treatment of different high temperatures. 

In addition, *WcHSP100-2* showed less expression than *WcHSP100-1* in all strains, and there was no significant change in expression within the same strain after treatments for various time points. Furthermore, when compared to other strains, gene expression in *W. cocos* ‘S10’ was lower at all-time points, reflecting it lower responsiveness to temperature changes. Moreover, the expression level of *WcHSP100-3* was inhibited when the temperature was above 35 °C. The expression levels of the *WcHSP100-3* gene in *W. cocos* ‘86’, ‘5.908’, and ‘S10’ strains were not significantly changed, but strain ‘5.78’ displayed a significant change in expression compared to control. Initially, the expression of ‘5.78’ was increased, but it decreased with extension in time period, implying that expression is influenced by temperature, but not significantly (Figure 4). In addition, the expression levels of these genes were relatively high in *W. cocos* ‘5.78’. Thus, combined with the heat-resistant analysis, we suggest that the *WcHSP100-1* is more important in response to high temperature stress.

**Figure 5:**
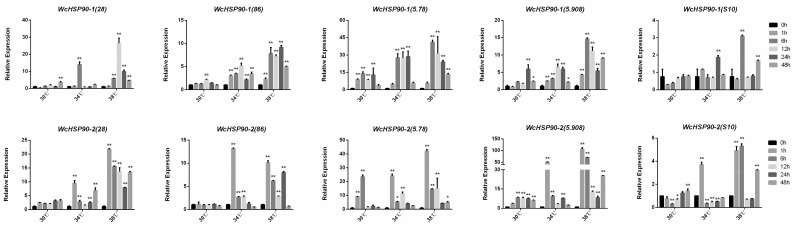
Analysis of expression patterns of *WcHSP-90* gene under different temperature treatments. The different strains ‘28’, ‘86’, ‘5.78’, ‘5.908’, and ‘S10’ of *W. cocos* are shown. All data are means ± SD of three technical replicates; the 0 h sample is taken as control, two-way ANOVA was employed to analyze the data, and significant differences are represented by asterisks: * *p* < 0.05; ** *p* < 0.01.

As shown in Figure 5, the expression profiles of the most *WcHSP90s* could be induced by the high temperature. Among them, the expression of *WcHSP90-1* was increased in different cultivars, then decreased with the increase in temperature and time, but not significantly. The highest and the lowest expressions were noticed in ‘5.78’ and ‘S10’ strains, respectively. Following 6 h of treatment at 38 °C, the highest expression level was observed, indicating that high temperature induces *WcHSP90-1* gene expression. Moreover, high temperature has a more significant effect on *WcHSP90-2*, which peaked at 1 h after 38 °C treatment, especially in the strain ‘5.908’ (Figure 5). In short, *WcHSP90-1* and *WcHSP90-2* showed similar expression patterns and displayed an increase in expression with increase in temperature.

**Figure 6:**
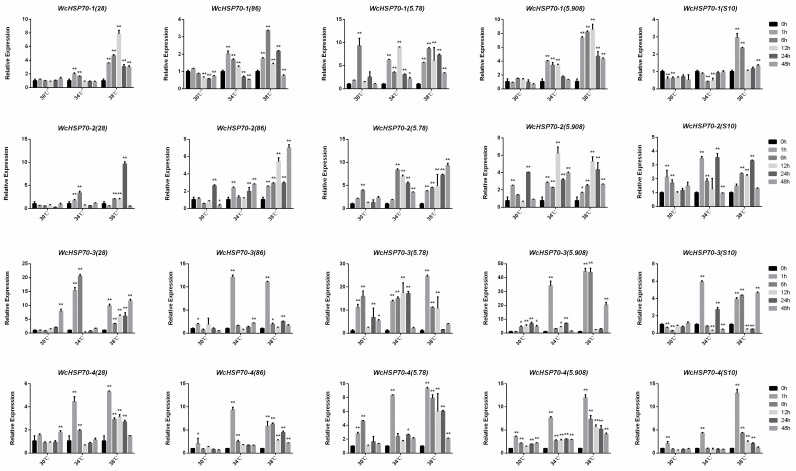
Analysis of expression patterns of *WcHSP-70* gene under different temperature treatments. The different strains ‘28’, ‘86’, ‘5.78’, ‘5.908’, and ‘S10’ of *W. cocos* are shown. All data are means ± SD of three technical replicates; the 0 h sample is taken as control, two-way ANOVA was employed to analyze the data, and significant differences are represented by asterisks: * *p* < 0.05; ** *p* < 0.01.

All the *WcHSP70* genes also showed an increase in expression with the increase in high temperature. *WcHSP70-1* and *WcHSP70-2* have the same trend of high-temperature response, which was upregulated and induced by high temperature, but the expression level was relatively low. These two genes showed higher expression levels in ‘28’ strains at 38 °C and treatment with all temperatures in ‘5.78’ strain, implying that the *WcHSP70-1* and *WcHSP70-2* genes are less sensitive to high temperature. On the other hand, *WcHSP70-3* and *WcHSP70-4* displayed a more obvious response to high temperature. *WcHSP70-3* displayed highest expression in comparison to other *WcHSP70* genes and gained maximum expression at 1 h of heat treatment. *WcHSP70-4* expression was low at first, increased significantly after 1 h, and then decreased, indicating that this gene is transient and can respond quickly to high temperature. What is more, when the mycelium was under the heat stress, the *WcHSP70-4* could keep continuous high expression in the heat-resistant strains ‘5.78’, ‘28’ and ‘5.908’, but did not remain high during the late phase in heat-sensitive strain ‘S10’ (Figure 6).

**Figure 7:**
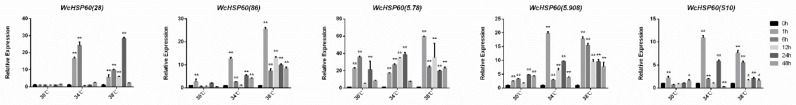
Analysis of expression patterns of *WcHSP60* gene under different temperature treatments. The different strains ‘28’, ‘86’, ‘5.78’, ‘5.908’, and ‘S10’ of *W. cocos* are shown. All data are means ± SD of three technical replicates, take the 0h sample as control; two-way ANOVA was employed to analyze the data and significant differences are represented by asterisks: * *p* < 0.05; ** *p* < 0.01.

The expression of *WcHSP60* was also tested and showed results similar to those of *WcHSP-70*, which increased with the increase in temperature, but later on decreased with time and showed the highest expression at 1 h (Figure 7). The expression level of *WcHSP60* was relatively higher in *W. cocos* ‘5.78’.

**Figure 8:**
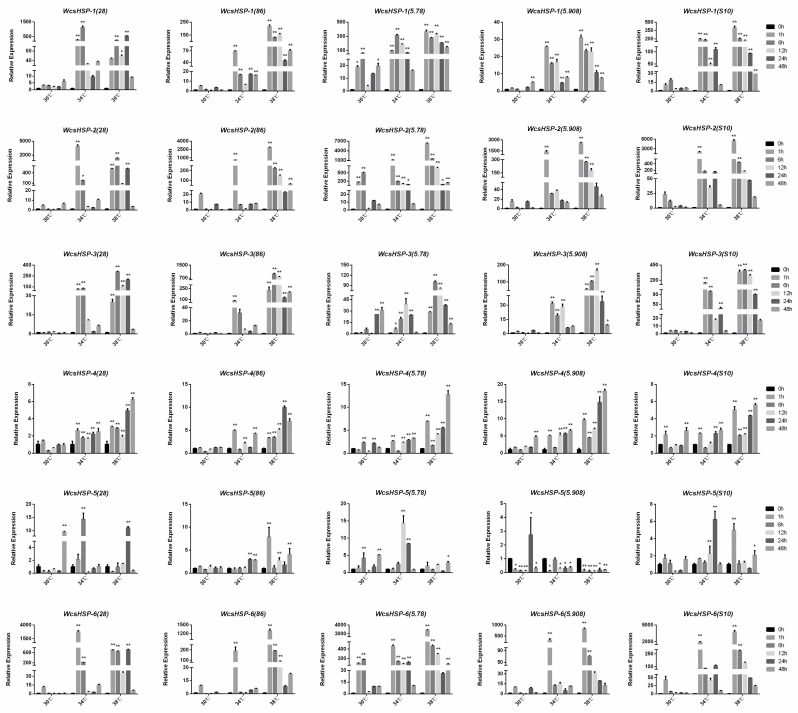
Analysis of expression patterns of *WcsHSP* gene under different temperature treatments. The different strains ‘28’, ‘86’, ‘5.78’, ‘5.908’, and ‘S10’ of *W. cocos* are shown. All data are means ± SD of three technical replicates; the 0 h sample is taken as control, two-way ANOVA was employed to analyze the data, and significant differences are represented by asterisks: * *p* < 0.05; ** *p* < 0.01.

*WcsHSPs* were reacting most violently and rapidly under the high temperature; in most cases, their expression could increase hundredfold before and after the heat treatment. *WcsHSP-1* showed the highest expression at 34 °C after 1 h of treatment, then gradually decreased with an increase in temperature, indicating lesser response to rising temperature. The expression level of *WcsHSP-2* increased significantly after 1 h of treatment at 34 °C and 38 °C and subsequently decreased with extension of treatment time. *WcsHSP-3* expression increased with increasing temperature (Figure 8) and peaked at 1 h and 6 h after treatment. More importantly, after 12 h at 38 °C, the expression level was also high, indicating good heat resistance potential. *WcsHSP-4* showed modest expression compared to other genes but increased with rising temperature and time, indicating that it can withstand high temperatures for a longer duration. The expression of *WcsHSP-5* was low and almost similar to that of the control. The expression of *WcsHSP-6* was high at high temperatures and increased with the rising temperature, with a peak after 1 h following various temperatures. Then, it dropped dramatically, indicating a transitory reaction to the high temperature. In short, *WcsHSP-1*, *WcsHSP-2*, *WcsHSP-3*, and *WcsHSP-6* had relatively high expression levels at 1 h while the expression of *WcsHSP-4* and *WcsHSP-5* was inhibited. Further, all the *WcsHSP* genes showed high expression levels in the ‘5.78’ strain.

### 3.6. Prokaryotic Expression and Thermal Function Analysis of Heat Shock Protein of W. cocos

**Figure 9:**
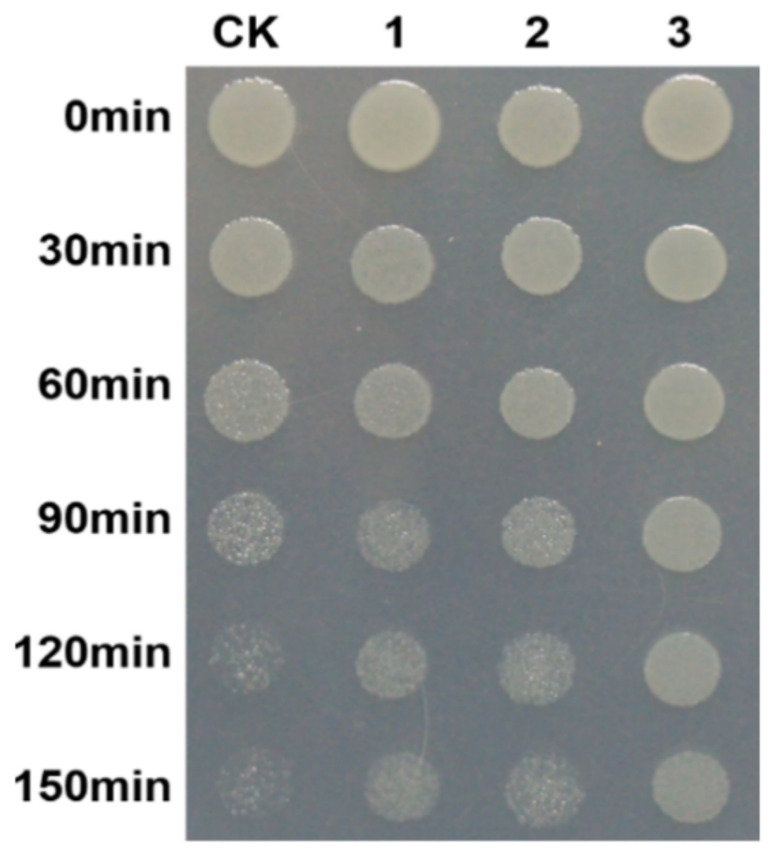
Growth of colony on the plate after heat stress. CK: control group pET32a (+), 1: pET32a (+)-WcHSP100-1, 2: pET32a (+)-WcHSP90-1, 3: pET32a (+)-WcHSP70-4.

Based on heat treatment of mycelial and the qRT-PCR results, *WcHSP70-4*, *WcHSP90-1* and *WcHSP100-1* were suggested to play important roles in response to the heat stress. Therefore, we cloned these three genes from cDNA extracted from strain ‘28’ for transformation in *E. coli* BL21. According to Figure 9, after 0 min, there was no change in the growth of *E. coli*. However, with the extension of heat shock time, *E. coli* showed a downward trend of growth, but the *E. coli* which expressed the WcHSP70-4, WcHSP90-1, or WcHSP100-1 grew better than the control. Especially after heat treatment for 150 min, the control group showed no growth, but the experimental group could grow slowly. Beyond this, we also observed that WcHSP100-1 could protect the *E. coli* against heat stress better. These findings imply that heat shock proteins play a critical role in high-temperature resistance and these heat shock proteins can help *W. cocos* to cope with the high temperature stress.

## 4. Discussion

High-temperature stress is an important environmental stress that affects the growth and development of fungi. Therefore, it is necessary to understand the mechanism by which fungi respond to high temperatures. Heat shock reactions in *Saccharomyces cerevisiae* have been thoroughly studied [23,24], but little is known about heat shock reactions in *Basidiomycetes*. *Basidiomycetes* are one of the largest divisions of fungus and contain many commercially important fungi, e.g., *W. cocos*. It is important due to higher amounts of bioactive secondary metabolites and it has attracted the attention of researchers. 

The hyphae of different strains of *W. cocos* were treated with different temperatures, and mycelium had the best growth state at 28 °C (Figure 1 and Figure S1), consistent with the most reported studies [25,26]. Although the mycelium could keep a stable growth rate at 30–34 °C, the density and color of hyphae point out it had been influenced by the high temperature (Figure S1). When the temperature rose above 35 °C, the mycelia growth was severely inhibited and began to browning, then stopped growing at 40 °C. These results showed that several strains of *W. cocos* had similar stress response and that may be caused by the similar climatic range. In any case, there still were some differences in sensitivity to high temperature between different strains; ’28’ and ’5.78’ strains had a better heat-resistance.

Previous investigations indicate that the reaction to heat stress is connected with the expression of heat shock genes. As a result, there is a rising interest in employing heat shock proteins to understand the molecular basis of heat stress response [27,28,29]. Therefore, we cloned the different *WcHSPs* from the *W. cocos* ‘28’ strains, and bioinformatics analysis were performed. Finally, we obtained three *WcHSP100s*, two *WcHSP90s*, four *WcHSP70s*, one *WcHSP60* and six *WcsHSPs* based on the transcriptome sequencing data and cloned them. In the present study, most of HSP family genes, including *WcHSP90s*, *WcHSP70s*, and *WcHSP60*, had highly conserved between the *W. cocos* ‘28’ and *W. cocos* MD104, whereas the *WcHSP100* family genes are different between them (Table S1). In addition, we also found that the *WcsHSP* family genes (*sHSP-1*, *sHSP-4*, *sHSP-5*) showed highly conserved, but others display the significantly differences between the *W. cocos* ‘28’ and *W. cocos* MD104. The results indicate that these HSP family genes have functional differentiation among the different *W. cocos* strains.

HSPs are synthesized in large quantities when an organism is subjected to high-temperature stress and act as molecular chaperones to protect the functional protein and cell in various stresses responses. Proteome analysis of *Asparagus bicarporus* also determined that genes of the *HSP70* family played an important role in resisting heat stress [30]. The studies also showed that high-temperature stress could induce the expression of HSP60 protein in *P. pellucosa* to resist the oxidative damage caused by high temperature [31]. In addition, *Hsp17*, *Hsp22*, *Hsp70*, and *Hsp90* genes of ‘*Glucoderma lucidum*’ hyphae were expressed in different degrees to resist heat stress [32]. Different kinds of edible and medicinal fungi produce different heat shock proteins. Furthermore, we investigated the expression of 16 heat shock proteins in different strains under different temperature treatments by qRT-PCR, and all genes were regulated by high temperature. The expression of most heat shock proteins was increased under heat shock and small molecular heat shock protein family genes showed the highest response. For example, *WcsHSP-2* had a much stronger response than the control, demonstrating that heat shock proteins are involved in *W. cocos*’ heat shock response. This result is consistent with the expression of heat shock protein genes in other edible fungi [15,33,34,35,36]. In addition, we also determined that *WcHSP70-4*, *WcHSP90-1* and *WcHSP100-1* had different expressions in heat-resistant and heat-sensitive varieties. These three genes might play a significant role and contribute to the difference in heat resistance of different varieties.

As research reported, HSPs expression is regulated by HSFs, which are central regulators of HSPs expression and participate in heat stress response [37]. Herein, we also found WcHSF3 could interact with *Wc*HSP60, *Wc*HSP70, and *Wc*HSP90 (Figure S2), and interaction among different *Wc*HSPs suggests that these can regulate or inhibit expression of each other [38]. *Candida albicans* responds to temperature via Hsf1 and Hsp90 to orchestrate gene expression and chromatin architecture, thereby enabling thermal adaptation and virulence [39]. Furthermore, we also analyzed the expression of *WcHSFs* under heat treatments; the results are shown in Figure S3. Through the trend comparison of HSFs and HSPs expression, we determined that the expression of *WcHSF3* had the similar change trend to *WcHSP70-2*, *WcHSP60, WcHSP100-3* and opposite trend to *WcHSP90-2* under different temperature treatments (Figure S4). Therefore, we conjectured that *WcHSF3* might be an activator of *WcHSP70-2*, *WcHSP60, WcHSP100-3* and a repressor of *WcHSP90-2*, as well as a significant regulator in increasing *W. cocos* adaptation in high temperature. However, the regulatory relationship between *WcHSFs* and *WcHSPs* needs to be further verified by more experimental confirmations.

Finally, in order to better verify the function of the heat shock protein gene of *W. cocos*, we performed the corresponding high temperature resistance of WcHSP using heterologous expression in *E. coli.* The results showed that the survival rate of *E. coli* containing recombinant plasmid was significantly higher than that of the control group and the heterologous expression of *W. cocos* heat shock protein enhanced the tolerance of *E. coli* to high temperature, suggesting that the *W. cocos* has a role in heat shock high-temperature stress tolerance. Our findings are consistent with prior findings, which revealed that HmHSP70 and HSP60 proteins can improve *E. coli* heat tolerance by expressing them in *Eugenia eentinus* and *Lentinus eentinus*, respectively [40,41,42]. *HmHSP70* heterologous expression in transgenic tobacco strains improved heat stress resistance. These findings suggest that the heterologous expression of the *HmHSP70* gene in the mushroom will also enhance response to heat stress. Moreover, similar results were reported about the *HSP100* gene [37,43]. In short, heterologous expression of heat shock proteins can enhance the tolerance of organisms to high temperature, indicating that heat shock proteins play important roles in high-temperature stress. 

In general, our findings provide insight into a better understanding of the functional characterization of *W. cocos* heat shock proteins, and lay the theoretical basis for studying the molecular mechanism of heat resistance in *W. cocos*. Importantly, *WcHSP100-1, WcHSP90-1* and *WcHSP70-4* were considered to be the key family genes for responding to the high temperature in *W. cocos*, but the function identification and regulation mechanism of *WcHSPs* and *WcHSFs* in heat treatment need further studies.

## Figures and Tables

**Table 1:** Physicochemical properties of heat shock proteins in *W. cocos*.

Name	Amino Acid Residue Base	Molecular Formula	Molecular Weight (kDa)	Isoelectric Point	Fat Soluble Index	Subcellular Localization
WcHSP100-1	904	C_4385_H_7195_N_1267_O_1354_S_23_	100.07	5.68	100.5	cytoplasm
WcHSP100-2	956	C_4688_H_7615_N_1383_O_1429_S_32_	107.24	6.53	91.59	nucleus
WcHSP100-3	1043	C_5144_H_8237_N_1447_O_1601_S_28_	116.87	5.31	95.68	nucleus
WcHSP90-1	800	C_4019_H_6315_N_1073_O_1273_S_17_	90.58	4.75	84.53	endoplasmic reticulum
WcHSP90-2	695	C_3512_H_5586_N_906_O_1116_S_18_	78.935	4.91	86.85	cytoplasm
WcHSP70-1	675	C_3252_H_5227_N_903_O_1042_S_13_	74.06	5.15	84.8	endoplasmic reticulum
WcHSP70-2	612	C_2940_H_4765_N_829_O_933_S_13_	67.07	5.43	87.53	cytoplasm
WcHSP70-3	780	C_3799_H_6073_N_1057_O_1187_S_17_	86.09	5.23	85.68	cytoplasm
WcHSP70-4	619	C_2945_H_4816_N_826_O_946_S_13_	67.35	5.42	89.18	cytoplasm
WcHSP60	456	C_2091_H_3444_N_578_O_674_S_14_	47.91	4.99	96.32	cytoplasm
WcsHSP-1	158	C_775_H_1214_N_226_O_246_S_3_	17.73	5.93	64.81	nucleus
WcsHSP-2	173	C_842_H_1321_N_247_O_267_S_7_	19.42	5.74	70.69	nucleus
WcsHSP-3	137	C_684_H_1079_N_203_O_210_S_4_	15.63	9.03	72.55	nucleus
WcsHSP-4	266	C_1398_H_2078_N_332_O_356_S_9_	29.52	6.2	110	cytoplasmic membrane
WcsHSP-5	287	C_1464_H_2201_N_357_O_385_S_11_	31.32	5.81	109.2	cytoplasmic membrane
WcsHSP-6	128	C_627_H_984_N_180_O_204_S_2_	14.37	5.38	75.39	nucleus

## Data Availability

The data presented in this study are available on request from the corresponding author.

## Supplementary Materials

The following supporting information can be downloaded at: https://www.mdpi.com/article/10.3390/bioengineering10030390/s1, Figure S1: Growth of *W. cocos* strains mycelium at different temperatures; Figure S2: Expression analysis of *WcHSFs.* Figure S3: Interaction network analysis of WcHSPs and WcHSFs. Figure S4: Co-expression analysis of *WcHSF3* and *WcHSP60*, *WcHSP70-2*, *WcHSP90-2*, WcHSP100-3. Table S1: Information of the *HSP* gene family members in *W. cocos;* Table S2: All primers used in this study. Table S3: Amino acid sequence of the WcHSPs and WcHSFs in *W. cocos* ‘28’ strains.
