# Supplementary material for: Bioinformatics Analysis, Expression Profiling, and Functional Characterization of Heat Shock Proteins in Wolfi-poria cocos
Source: Bioengineering (Basel). 2023 Mar 22;10(3):390. doi: 10.3390/bioengineering10030390 (PMC10045903; doi:10.3390/bioengineering10030390)
Supplement: Supplementary file 1 [file bioengineering-10-00390-s001.zip › bioengineering-2230986-supplementary.pdf]

Supplementary Materials

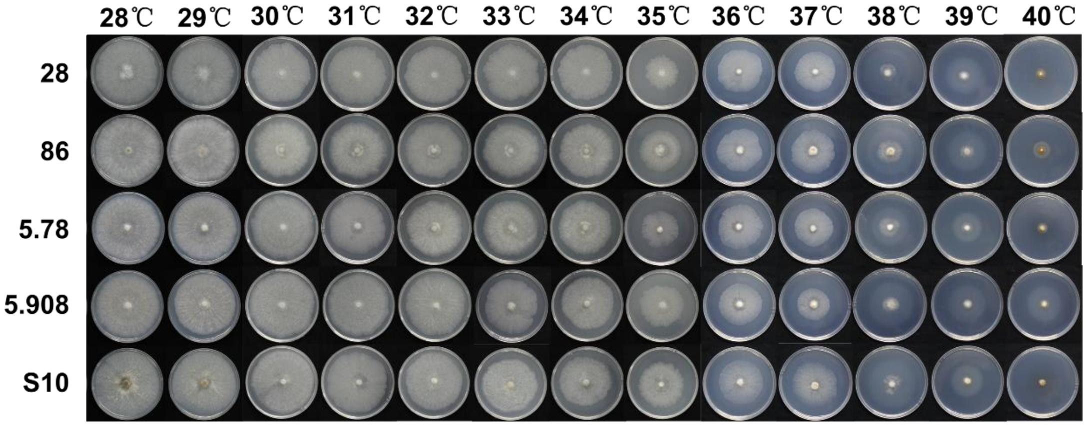

Figure S1. Growth of W. cocos strains mycelium at different temperatures

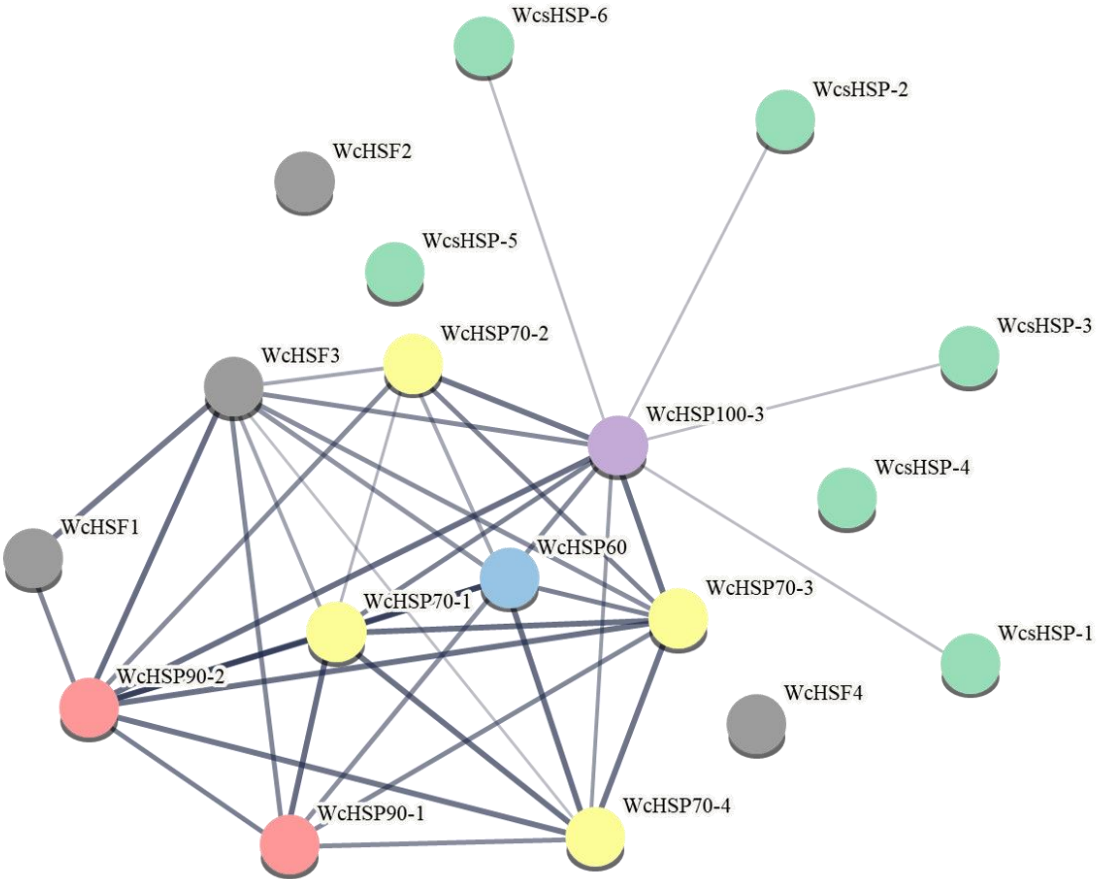

Figure S2. Interaction network analysis of WcHSPs and WcHSFs by prediction. Black, green, blue, yellow, red, and purple denotes WcHSF, WcsHSP, WcHSP60, WcHSP70, WcHSP90, and WcHSP100, respectively. For prediction of protein-protein online program STRING (https://string-db.org/) was used.

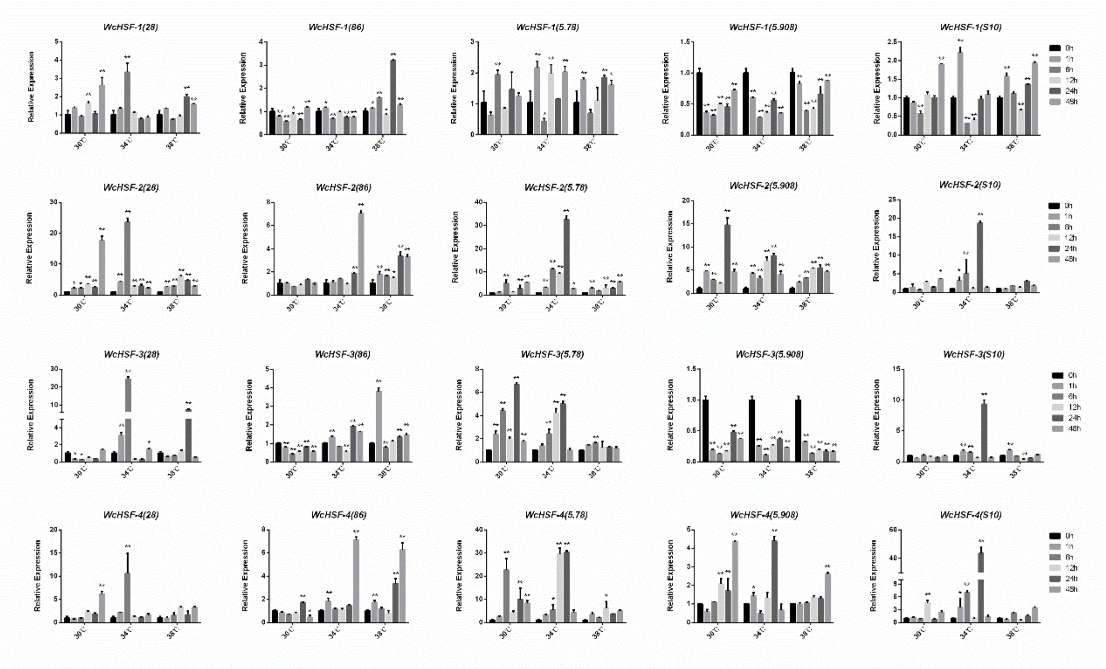

Figure S3. Analysis of expression patterns of *WcHSFs* gene under different temperature treatments. '28', '86', '5.78', '5.908', and 'S10' are different strains of *W.cocos.* All data are means ± SD of three technical replicates, take the 0h sample as control, two-way ANOVA was employed to analyze the data and significant differences are represented by asterisks: *, $p < 0.05$; **, $p < 0.01$.

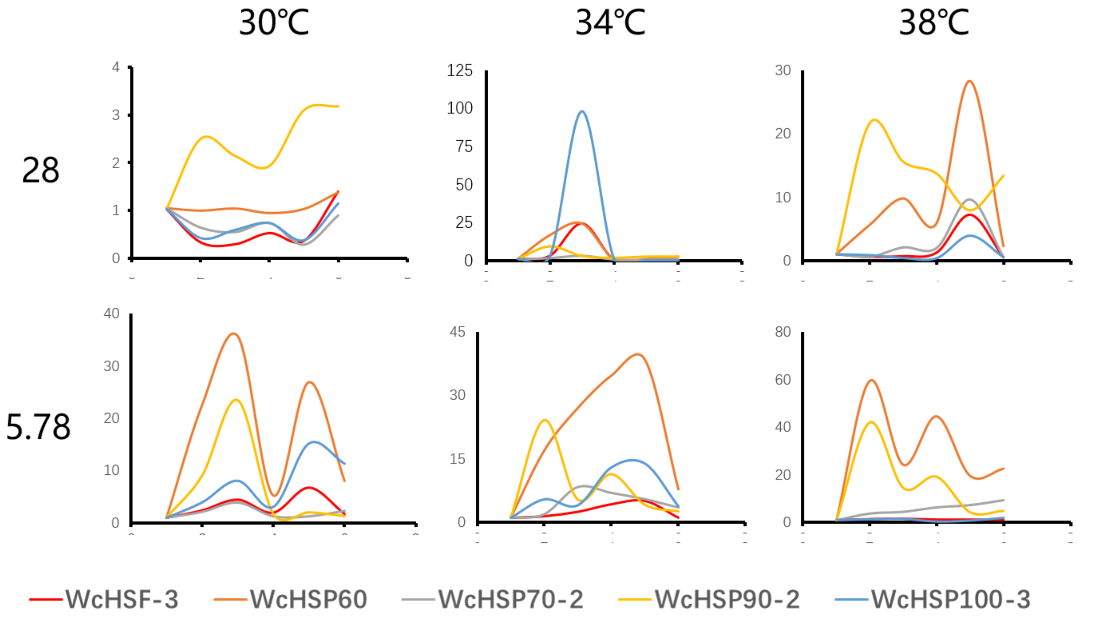

Figure S4. Co-expression analysis of *WcHSF3* and *WcHSP60*, *WcHSP70-2*, *WcHSP90-2, WcHSP100-3*.

Table S1. Gene information.

Classification	Gene name	Gene ID
HSF	*WcHSF-1*	Cluster-960.3075
	WcHSF-2	Cluster-960.3449
	WcHSF-3	Cluster-960.3695
	WcHSF-4	Cluster-960.4433
sHSP	*WcsHSP-1*	Cluster-960.3159
	WcsHSP-2	Cluster-960.3342
	WcsHSP-3	Cluster-889.0
	WcsHSP-4	Cluster-960.3369
	WcsHSP-5	Cluster-960.3768
	WcsHSP-6	Cluster-960.4799
	TpsHSP-1	OJT14810.1
	MD-WcsHSP-1	PCH42765.1
	MD-WcsHSP-2	PCH42744.1
	MD-WcsHSP-3	PCH38688.1
	MD-WcsHSP-4	PCH38030.1
	MD-WcsHSP-5	PCH36592.1
	MD-WcsHSP-6	PCH34468.1
	MD-WcsHSP-7	PCH34056.1
	GfsHSP-1	OBZ74880.1
	LesHSP	GAW02286.1
	ScsHSP	AAA79010.1
	HmsHSP	RDB20963.1
	LesHSP-1	GAW02286.1
	TmsHSP	KAF8237894.1
	PnsHSP-1	PAV22864.1
HSP60	*WcHSP60*	Cluster-960.5154
	TpHSP60	OJT02927.1
	GfHSP60	OBZ72799.1
	LeHSP60	GAW09714.1
	ScHSP60	AAA34690.1
	HmHSP60	RDB24487.1
HSP70	*WcHSP70-1*	Cluster-960.3470
	WcHSP70-2	Cluster-960.3529
	WcHSP70-3	Cluster-960.5228
	WcHSP70-4	Cluster-960.6601
	MD-WcHSP70-1	PCH35569.1
	MD-WcHSP70-2	PCH43031.1
	MD-WcHSP70-3	PCH38384.1
	MD-WcHSP70-4	PCH35950.1
	TpHSP70	OJT15625.1
	GfHSP70	OBZ69999.1
	RsHSP70	CUA66922.1
	LeHSP70	GAW04349.1
	ScHSP70	AAA35099.1
	HmHSP70	RDB25126.1
	TmHSP70	KAF8235873.1

	PnHSP70	PAV24310.1
	OsHSP70	EQL03305.1
HSP90	*WcHSP90-1*	Cluster-960.2162
	WcHSP90-2	Cluster-960.4365
	MD- WcHSP90-1	PCH41165.1
	MD- WcHSP90-2	PCH38044.1
	TpHSP90	OJT15706.1
	RsHSP90	CUA70109.1
	LeHSP90	GAW05303.1
	HmHSP90	RDB24182.1
	PnHSP90	PAV23670.1
	OsHSP90	EQL00771.1
HSP100	*WcHSP100-1*	Cluster-960.3653
	WcHSP100-2	Cluster-960.3911
	WcHSP100-3	Cluster-960.4077
	TpHSP100	OJT15823.1
	RsHSP100	CUA76670.1
	LeHSP100	GAW03650.1
	PnHSP100	PAV22376.1

Table S2. Amino acid sequence of the WcHSPs and WcHSFs in '28' strains.

Gene name	Amino acid sequence
WcsHSP-1	MSLSSFFYEPFYTLADFDRLFDEAFSARTGPRGQSADRQVQRQDSSTRFLRPRMDLHEDAQANAVTATFELPGLNKENVNIDVHNGVLTVSGEAQVQSDRDEHGYAVRERRYGKFSRAVPLPQGVKSEDIRASMENGVLTVTFPKTTPETAPKKITIS
WcsHSP-2	MSYPHFFYDPFAEFNRLLDDALTERNVGYPQGQAKYSAAPIIAALHGLGEREPQGPELSAHIDITRCRMDVHENSQTNQVEATFELPGLRKEDVSIDVHNNRLTVSGESKQSTERNEAGYAVRERQYGKFSRTLQLPAGINTNDIKASMENGLLTVSFPRAAPQEGPKRITVS
WcsHSP-3	MSLTSFFYQPFYSLADFDRLFNEAFSARTNPANTNGDRQVQRQESSSRLLRPRMDLHEENVVTATLELPGISKENVQIDVYNGVLNVSGESRLFSDRDENGYAVRERRYGKFSRAIPLPQGIKVSCAPQRKVKNCIP
WcsHSP-4	MANHALNLNPPNADLHISTHGSDWLWAAFSVFAFSLLVMIVLDLLRPRGTRLFHQLAVIILATFTIGYFSMASDLGATPIAVEFRGHGSDPTRQIWYVRYIQWFITFPLSLLEVLLATGLSLSDITTTLFMAIVVVVCGLVGALVHSTYKWGYYVFGVAALFYIWYVLLWHGAQTTFPGGGVLRPGYLRSAGFLAFMLITYPICWACSEGGNVISNTSEMIWYGILDILAGPGFLFFFLWHLRDVDYATFGLSSGKYTDTAEKVGA
WcsHSP-5	MAGNQAVNINPPNANENLTKQGSDWLWAAFSLMSLSLLVAVVVTFLRPRGTRLFHQIAVVVLAVSSITYFSLASDLGATPIAVEFRGHGGDPTRQIWYVRYIQWFINFPLLLLELLLATGLPLSDIMTTLFMAIVVVVCGLVGALVHSTYKWGYYVFGCTALIYIWYALLWHAPSSTFAAGGVVRRGYYLAAGYFAFMLITYPICWACAEGGNVITVDSDMIWYGILDCLTIPVFLAFFLWEIRDVDYNAFGLHSGKFVAHNAASAKAAEAGEAAPVAASEAGAQQS
WcsHSP-6	MSLSRFLYEPFYSLADFDRLFDEAFSSRTNGGSDNTAVSRTLRPRMDLHQDEKANTVTATFELPGLKKEDVEINVHNNVLTVSGESNISSDRDENGYAVRERRYGKFSRALSLPQGIKVSGAYLSIVD
WcHSP60	MDLRRGSQAAVDRVVAFLAAHTKTITTTAEIAQVATISANGDTHVGNLIAQAMEKVGKEGVITVKEGRTIDDEIEITEGMRFDRGFISPYFVTDVKSQKVDFEKPLVLLSEKKISRLQDVIPALEAAAQARRPLLLIAEDVDGEALAACLVNKLRGQLQVCAVKAPGFGDNRKSILGDLAILTGAQVFTEDLDMKLEQLTPDMLGSSGSITITKEDTIILNGEGSKDAIQSRCEQIRSLIDDRTTSDYDRTKLQERLAKLSGGVAVIKVGGSSEVEVGEKKDRYDDALNATRAAVEEGILPGGGVALLKASLALASAPGSKPASTAPDAKPIPTANFDQDLGVSIIKRALTYPSRTILKNAGEESSVIVGTLLANYGSPDKFSWGYDASKGEYVDMIKAGIVDPLKVVRTALVDAAGVASLLTTSEACVVEAEEKDKGGAGAGMGAGMGGMGGMGF
WcHSP70-1	MSLPRRPTRRQSRRVVSSLSLAFFAFLALFLLCPAATANEDKRSEYGTVIGIDLGTTYSCVGVQRGGRVEIIANDQGHRITPSWVSFSDEERLVGDAAKHAFHSNPENTVFDAKRLIGRKVDDPEIKRDQKHWPFKVVSKNDKPAIQVKHRGEARDFTPEEISAMVLGKMKETAESYLGKPVTHAVVTVPAYFNDAQRQATKDAGTIAGLQVLRIINEPTAAAIAYGLDKKGGESQIIVYDLGGGTFDVSLLSIDDGVFEVLATAGDTHLGGEDFDNRVMDYLIKQYKKKTGTDVASNLRAMGKLKREVEKAKRTLSSQQSTRIEIESFENGNDFSETLTRAKFEELNMDLFRKTMKPVEQVLKDANLKKEDIDEIVLVGGSTRIPKVQQLLKEYFGKEPSKGINPDEAVAYGAAVQGGILSGDENLGDIVLVDVCPLTLGIETTGGVMTKLIPRNTVIPTRKSQIFSTAADNQPTVLIQVYEGERSLTKDNNLLGKFELSGIPPAPRGVPQIEVTFEIDANGIMRISAVDKGTGKSESVTITNEKGRLSPEEIERMVKEAEEFAAEDEAQRKRIEALNSLSSFVYGLKTQLGDQEGLGGKLNDEDKKTILDAVKETTDWIDDYGQSATAEDLEEKLADVQRTVNPITSKLYSGGGTDYSAGAEDEDPIHSHDEL
WcHSP70-2	MEDVFDGAIGIDLGTTYSCVGVWQNDRVEIIANDQGNRTTPSYVAFSAEERLIGDAAKNQAAMNPRNTIFDAKRLIGRRYDDPDVKKDMIHWPFAVVEKDGSPLIKVEYLGEEKTFSPQEISSMVLTKMKEISEAKLGKTVKKAVVTVPAYFNDSQRLATKDAGAIAGLDVLRIINEPTAAAIAYGLDRQSSAEKNVLIFDLGGGTFDVSLLNITGGVFAVKATAGDTHLGGEDFDNNLLEHFKKEFQRKTKLDISEDARALRRLRSACERAKRTLSSVTQTTVEVDSLFQGEDFSANITRARFEEINASLFKSTVEPVERVLKDAKMPREKVDDIVLVGGSTRIPKIQSLVSEYFGGRQLNKSINPDEAVAYGAAVQAAVLTGQTSDKTADLLLLDVAPLSLGVAMQGDIFGIVVPRNTPIPTNKSRTFTTVEDNQTTVTFPVYEGERTQCRDNRLLGEFELTGIPPMPRGQAELVTTFEVDANGLLKVTAQDRASGRKAQISITNSVGRLSSAEIEQMIKDAEQFKQADKDFSARHEAKSDLESYIHQVENTITSPDLGMKLKRGAKAQVEAELARALEKLEIEDSTADELRKAQLGIKRALQKATAGIR

WcHSP70-3	MAVVGIDLGALHSKIGVARHRGIDIIVNEVSNRATPSLVSFGVKQRAIGEAAKTLETSNFRNTIGGLKRLIGRTFQDPEIQ<br>EVEKKFINATLIDVGGTVGVQVQYLGEQRQFSITQVLAMYLGKLRDTAANELKSGVTDIVITVPGWYTDVQRRAVLD<br>AAQIAGLNVLRLINDTTAIALGYGITKSDLPEAENPRHVVFVDVGHSSTSCAVVAFSKGQLTVKSTAYDRHAGGRDIDY<br>ALVRHFASEFTDKYKIDVLSSPKATFRLAAGCDRVKKVLSANAEAPLNVESIMNDVDATSRLSREEYERLIAGVLDRIP<br>GPLQQALADSGLTIDQIDAVELVGGCTRIPAVRAKIQSVFEGKVLSTTLNQDEAAARGATFACAMLSPVFRVRDFSMHD<br>ITPYSIKVQWERQPDDTDDDTELVVFPKGNSIPSTKVLTFYRKQAFDIQAQYADPAALPGGINPWIARFTAKSVGPDENG<br>DFACVKLKTRLNLHGVMSFEAAYVEEIEEKEETMQVDGEEAPKKKKIVRKKDVPFVWAGTSLDVSVLDKFKEQEAQ<br>MHAADKLVKDTEDRKNALEEYVYDTRGRLDERYASYVKADEKERLLAALQDAEDWLYTEEGEDATKSAYVERLDA<br>LKKLGDPITFRYREAEERSRVVAQLRETINSYMGQATSGEERFAHIDAADLQAVVEKCATVQKWLEDQIARQAERPKD<br>VDPVLTSADVMKKRDEIIYFATPILTRPKPKPPKVEQPAGTETPKSGQETPNPPPQGEAAKEEPAAANEPPEMDVD
WcHSP70-4	MEGKTSRVIENSEGARTTPSVVAFTKHGERLVGLPAKRQAVVNSQNTVFAFKRLIGRQYSDAEVKKDMEHWPFKVVA<br>KPDGRPAVEVDNGGKKQLFSAEELSSMVLVKMRETAEQYLNKGVKHAVVTVPAYFNDAQRQATKDAGQIAGLDVLR<br>VINEPTAAALAYGLDRVDNAVIAVYDLGGGTFDISILEMQKGVFEVKSTNGDTHLGGEDFDIVLVQHILNEFKKESGLD<br>LASDPMAIQRIREAAEKAKIELSSTSQTEINLPFITADASGPKHINLKILRSHFESLVSPLIQRTIEPCKKALVDAGVKASEV<br>NEVILVGGMTRMPRVVETVKTIFGREPSKGVNPDEAVAIGASIQGGVLAGNVTDILLLDVTPLSLGIETLGGIMTKLIQR<br>NTTIPTKKTQTFSTAADGQTAIEVKVFQGERELVRDNKLLGNFNLTGIPPAPKGVPQIDITFDIDADGIVHVTARDKATN<br>KDQSMTIASSSGLSDKDIERMVADAEQYAESDKERKLVIEEANKADSVCHDTERAINEFKDQLDGAEKDKVTKLVAEL<br>RELAVKGQAGDASITADTIREKLNETQQASLGLFQKVYEKRNAEANASESTTSEEKKEEKKEEKKD
WcHSP90-1	MRFFRPLLLTLSVLASGVFAQDATDVPKQKHDYQSDVARLRKIVINSLYSHRDIFLRELISNANDALEKLRLTSLTEKEV<br>LSGAEEMNITIKALKDDDGTGRIIITDTGIGMNPEELTTNLGTLARSGTSEFLARAESTDTTGAGNLIGAFGLGFYSSFLV<br>ADRVYVASVAAKSSKNPSPAQYVFESGADESSFEIYADPRGNTLGRGTEITLVLKPDALEYTDPQRITELVDKHSSFSSVF<br>PIYLYTQRTEYVPVEEDEAEKKVENDAEEVDEDEATVEDAEDETKSQEPKMKEVTVDEWIHMNSQPPIWMRDPKTVS<br>DEEYENFYQATFKDYEKPLAWHHFSGDSGSGVSFKAIIYVPSHLDESYWQNPLLTNSRDIRLMVKRVFITSDLGEDALP<br>KWASWVKVVVDAEDLPLNVSREMLQSTRFLKQLRSIILKHLLQVLTRTVEEDPEKWTQVQSVYGNVFKLGAVEDVK<br>NRDKLVALTRFSTNQRNSTSLDEYLENKKKGQKQIFYLADMGSTTDHLAKSVFIEKLHARGYEVLLLTDPLDEIFVQNL<br>RVWKRVSFQDVAKAGLRFGDEDMSPEEEKEEQKALTEEFKPLLDWLKKEAKDVVRDVVISNRLVTSSCAVVADMMG<br>YTANIEKMMSSSHSGVRNPMHEFAKKQKIMEINPRSPTVQGLLRRVEQLPPEEEGQDLEAEEELREVASVLIDGALVRS<br>GFSVPDSNEFFVRVDRILRRSLGVSETAPTDTTVKPAPPVDPEPIEVEDPFQAEWPDYIPGSLRQPPQHEEQLVWEVEEID<br>DEGNPVKHDEL
WcHSP90-2	MASESFGFQAEISQLLDLIINTFYSNKEIFLRELISNASDALDKIRYSSLTDPSVLETGKDLVIRIVPNKEARTLSIYDTGIG<br>MTKADMVNNLGTIAKSGTKGFMEALSSGADISMIGQFGVGFYSAYLVAERVQVISKHNDDEQYIWESAAGGTFTITAD<br>TVNPPLGRGTEIRLYLKEDQLEYLEEKRIKDIVKKHSEFISYPIQLAVVKEVEDDEEEAKEEESEDKPKIEEVEDEDKPKK<br>TKKIKEKEIQNEELNKTKPIWTRNPSDITAEEYAAFYKSLTNDWEEHLAVKHFSVEGQLEFKAILYIPKRAPFDLFETKK<br>KRNNIKLYVRRVFIMDDCEDLIPEYLNFVKGIVDSEDLPLNISRETLQQNKILKVIRKNLVKKCLDLFTEIAEDKDNFSKF<br>YEAFGKNIKLGIHEDSQNRSKLAEFLRFYSTKSSEEQTSLKDYITRMPEVQKNIYYLTGESLSSVKDSPFLEVLKKKGFE<br>VLLLVDPIDEYAITQLKEFDGHKLVCVSKEGLELEETEEEKNAREEEAKQFEDLCKTVKDALGDKVEKVVVSNRITDSP<br>CVLVTGQFGWSSNMERIMKAQALRDSSMSSYMASKKTLELNPHNAIVKELKKKVAEDKADKSVRDLTYLLFETALLT<br>SGFSLDDPTSFAKRIHRMISLGLDVDEEESAPAASSSDDVPPAAEGASTSAMEEID
WcHSP100-1	MASSFNFTDKTQETLAAAIQLAKDYANAQVYPVHIAFALLNEGAGEQVPGGVNQQSGHSLFRSVIERAGGDTTAVKRS<br>LQKLIVRMPTQQPPPDEISLSSAALKVLRDAESLRKTMHDSYIAQDHLLAALIKDSTVAPILKEANLTEATLKTAIDQLR<br>GNRRVESKNAEQGFDALQKYAVDLTSLAEEGKLDPVIGRDNEIRRVIRILCRRTKNNPILLGEPGVGKSAIAEGLAQRIV<br>NRDVPASLLGRLYSLDMGALMAGAKYKGEYEERIKSVLNEIEKASDEGTGVILFIDELHLIMAGRGAEGGGMDAANL<br>FKPLLARGKLRCIGATTLAEYRKYIETDAALERRFAQVLVNEPSVPETISILRGIREKYEVHHGVRILDGALIQAASLAHR<br>YLTSRRLPDAAIDLVDEACASVRVTRETAPEAIDKLQRRKLELEVEIHALEREKDEASKERLALARKAIADVDDQLQPL

MAQYEAEKARGDEIQNVRRKIDELKAKADEAERRYDLATASDLRYYALPDLQARLAQLEAKKAEEDVQAGVGSDTV
TPEQIAEIVARWTNIPVTRLMSTEKEKLLKMERILGEQVVGQPEAVKAVANAIRLSRSGLRNAQRPIASFLMAGPSGTGK
TLLSKTLATLLFDSPDAMIRIDGSEYSEKHSISRLIGAPPGYVGHDQGGQLTEYIRRKPYSIVLIDEIEKASREFVTLFLQV
LDDGRLTDGQGRIVDFRNTVIIMTSNLGAAFLNDMGEGAVKSETRELVMGAIRAHFPPEFINRIDEIVIFRTLSQRNVLKI
VDLRLKEVQERLADKKMVLDIEPAAKQYLMSSGYSPIYGARPLNRAIQTELLNPLSVMILSDRIRDGETIRVRFDGPHN
RLAIIPNHEAGADSMDIDQDDIEDIEIEEMD

WcHSP100-2

MMADVQMDIPKYTIEYGRIIERTYDIAKEVQSSKVTPFHLLLALLCGKELNLHNLENFVPDNPSLFWSALEDLEPEHGS
AELVTTLWHVIQNEASKMDQPTSNAATSDGPAKLTRSRFRKILSREVNQSHSNSRIGGTGTIEPSEELIAVFGRANPLVSI
SGDDFAGPYHLLLSVVGDDGVSNFLKDSQIDRQEFVRAVRRRRPPKITSKDDESFPHLTKYATNMCDMAREGKLNEIIG
RDEEIRRVIQILSRRSKNSAVLLGEPGVGKTAVAEGLAHRIVKGEVPQSLRGTLWSLDLGALFAGAGKGEYELRVKNVV
EDVTRSQQSQSPAIIFIDELHLITVGKSPGGQNSGMDAANLLKPALARGQFRCIGATTLSEYRQHIEKDGALERRFAPVIV
DEPTIDEATTILRGIRERYEKHHNVWIMDQAIVTAVKLAHRYLTARRLPDSAVDLMDEACAAARVAQDMKPEAVDTLE
QEISHVDAHIRALERDTHPDDEEALTEAKKRRVELEGKMKQLLRQQQNLKQWWEQMSHQRTLLQKKRDEIKRLGDG
ASRTTANRLKDELRDLRGGLMALERQGPEIIDSISEFFGKNRHVISRSSPDIITPESIAEIVERATKIPVQRLLANEKSHLLQ
LESVLSQQVVGQPEAVKAVAHAIQVSRTGLGNTNRPIASFLFTGPSGTGKTLMSKALAKELFGQEMAMVRIDGSEYSE
GHSVSRLIGSPPGYVGYDQGGQLTEYVRRTPFCIVLLDEVEKACSEFLTLFLQVLDEGRLTDGQGRLVDFRNCVIIMTSN
VGAEILSTTSGEITRSTREQVLQRFKDVRFPPEFMNRIEEVVMFRTMSNEVMREILEKHLRELMQREGLKRLQGLDLDE
NAKNWLIQVGISPHYGARPLARMIQRELLNPLSRCLLEETVHEGDLVLVRCKPERDGLFIKSNREPAPPQHNGRNVGLR
LRGS

WcHSP100-3

MSKTGSVSSSHISLHETSPPTPQLKSLISDELQQILSCSVQLADDTSNNYSSLHIHPLHIALALLYEGLTYRTSPWLHEPTK
TWHVYPWERSLFWLVIGKAARNTLPISLDEHICHVTHPDRDTFIVQNSVGQAIWNKVEGLSDHDLQATGDQDDDPTY
QSSRWNDSTRGLLSRAKEICHDKADEFIAPHHVLLALLKDALVREILESQDLDPNILEDQIKGLRQRNITRSESLPRFSLL
NQFATDLTEEIYDKIKRNELIDPVIGRNYEVRHIIAILSRCSKNNAVLLGDPGVGKTAIAEGLAYRIVKQEVPESVVARVF
NLDLGALLASTACKSAYERIVELILQEIAEHEERGIRVILFVDELSQITIGGWRDDGTGLDAATLMKPFLSKGKLRCVGC
STFEDYRDSVEKDAALSRQFSAVFVCESTVPETVAILRGMKKGLEQHHNVVILDDSIMAAASIAAQYFTHKRLPDAAID
LVDEACAALKTAGNEQREKRMQLQRRQVLLEMDIASLEREYDEDSTVKLTQALAQLSQVQQQLQQISISGERSASINS
KLEELNVQIKQKERDMLQYGKAGQRAERLKCVQELWKLKDARAEEMSALERLQSKQLDNVDESQRQTNDHASNSE
EAKIVTKRDIVQAASWYRPVHCSTWKNAKQDALAKFLETLPKMVIGQEEAVDAVSSAIRCMMSGLTDPTRPVASFIFG
GPAGSGKTFLAKELAKALLGSTSKLVSINAGDYNAPHTLTRLIGTPVCTGFDHGGQLTECVRRKPFSVILIREIENACPEF
RLYIQRILDEGSTKDGAGKAVDFRNCIVIITTGIGQINSKATFDEDEERKHYLNEINEWFSVEFLARIDELVVFRRITADIIG
HIIAARLEEISKQLTRLKLHLTVDDEVQWYLEDQAWSADSGARHLEKVIRDEIIHPLAIRLLDDRDIENWRVRLSIEEYD
DGITIELLEPLPPVDLQSSGGSSDTETTTAPSPSASVNSLLESYEPRQPCDSVTTGSLEPDQSVVPTESFIGSTFRPPIPQFRP
QRLF

WcHSF-1

MMGAYPDGDVARASLAQMTEISRRAEVAGMTFSATPASRQQGSQGPMSRIMAAAGLNVDAPVRPMSRQNALARIEEL
ARNRPPSSQGQPAASGSGAGAVPAAQPQASGSGAAAGAGAGEGAFDAEAFLLGDAGSVGNYAPGTMSHEGLQVFTV
GHLMPKSTLEDANGNWAFDPNALAGVVMPTPQGMGGDPEQEGEGERKGPYPIASFAEGEAGTSAATSGRPGSAQKL
RVRRSTYVPGWAVPPKVLLVDDDAVSRRLSSKFLQVFGCTIDVAVDGVGAVNKMNLEKYDLVLMDIVMPKLDGVSAT
SLIRQFDHMTPIISMTSNSKPNEIIKYYSSGMNDILPKPFTKDGLLDMLEKHLMHLKVIQTMQRVPRSVGIPPLNDAGFD
QALAMQPAQSAGGFTLGDDDGKINPLAGMGLTDEQYTLILQNLVSGDSFMGVIGGIEESASGKRSLDDSSDGRDAKRS
RFEIVE

WcHSF-2

MYVATPGFAPLLSAFQIYGFMRKVNLRNVDPAIDDPDASTWSHPTLNRHSPPEVVANFKRRVPPRVPKPRKRDNEVPQP
LPSARPQLNIGPMGMAQQPGSPRQGARARGFSAPGSFTPLTQPGAPGWGTSYPRSTLPPLTVPEPPMSHASMYSQHSA
HPLHPITPTDDAPTSSFTSPMSYTNSSRETMMLPSPNGQYPYASDSATSWAFSPTSAASSHSSGSLSSLLNPSGSVSSAYS
NSSRPTPPQINTYTSYNGVQMSRGHHSAAPLSPESRPTSGYSSSMSSLPDPYAESPNHYSHHDYSRPSSSHHQQQGRPLT

	PSSSRPPSSKSYNNAGSLSIRRARRHSQAMQPYPSPYAEHPPLSAGSDRPSSSPQPGDEHSSAIPRVRSMIQLPTVDGYGF<br>SSSQSEFAYAAVDDGHAHQQQQQQHQQQQHLYGVSGRSVRPSTSASSLSTASSAANTPGEFAPGTGEADINRCEYHSC<br>CAAGRRRDACGLRYGGPGDHVADCVPSYSRAHTVSPDFGFVQMNEHIPQYAKAGEM
WcHSF-3	MDAIMQCPVLNHERFAREVLGRWFKHQKFTSFVRQLNMYGFHKIPHLQQGVLKSDTDTEPWNFEHPHFHRGQPDLL<br>CLIQRKKQPANSTGDEPDIVDSHQSAPLAGNASQVLDINSIVNGIAAIKRHQQAISADLNALKKSNDLLWEEATLTRQR<br>HDKHQDTINRILKFLAGVFGNNTESINKNESGRPPSVVPRARQRLLIGDGGFPKGKSVEIADVDEDDEEVHMAHPQEG<br>NKTPLAGKLPHDILQHTIYELYRLAGQTAAVEPTVESPMMSPSPSAPPSEPFSTVSVDSTASGYTARPVTRPSEVLSRTST<br>SNNSNFGSATEVPTRTTFTQNAARADATILPTALTSFTPNAANASYSDNIWQTAFQQLLNNPLQMQRLMQAIAAQNSNI<br>PPPDNSSMIHSQPSAQIAPYDQTYNDYNRYRNDLPVAAPPSQSSLPMLSAISNDAPPLEPLLHENNARLQKTYRDAVEIE<br>ADMDVLQHSLNSLIHDLGMDPQVLIAQAQERDPPTGNIPGALLSNGGHANGVHANGFRPNEMDSDALMLGVAANGH<br>QEEAAPDLFLESLLNGMSPGTDGNMEYSDVTDHFNPSARIDGITVADASTEQLAAFLDEVSDTASPHVRSPEIKTTPAK<br>RKSDVAGLAPSIVDVEKTAAAGPKSKRKR*
WcHSF-4	MMGAARVLGLIEGRRARKGRTPNLRLCHRPQIVPSHALCMLSSPREPMDAQYAPMPDHHFAPPAHAQWSQAAHLLPP<br>PAPAPFPSPASPAPSLADQHYASYYSHPPPAPQHGLGDSAVVRQIDRSSLSLNLSSLSVASPTNLSPINPSPHPSTQPSSHVS<br>PITPISPAAACALGPAHAHAQHLGQSFSFAPPEPGVRYDDAPQSSYDYTAGPARRGLSSRSSSASDKSVPRKRSFPPGAL<br>APSVEEQFDPAPADAGYDDVDMTYGALDAHSPVDGSTSGGEADDPMRAPDPPQALPPVGMLGKPLGTNNFVTKLYQ<br>MINDPKSAQFINWTDLGTSFVVSNVGEFSRTILGSHFKHNNFSSFVRQLNMYGFHKINRTPRAQRTSADVQTWEFSHH<br>KFLRARPDLLEEIKRKALEPDPSVKQRVELPGEVAAQLREVREANHRLAGALHAERRRVARLAGLTKTLWDAVARSWP<br>GSVPGPFPNDLLDAAESPNIYITSPSAHGGPPQHPHFALLHPLSPGASPTAPEFPARFAHAHYDVHAPGLSAARYETALA<br>TPLPPSPGPAGAGAGEPGEFGEDARVGAKRARTEGEGARRGTRARSDSAPLGYGLGAGWAGARPRSGSGLAVSAGGG<br>QCASPSSGLAQGGMGALGFGRAGARREEVQSIGALSRQGGGQGQGQGQGLPMLAIPGK

Table S3. Primer sequences used in this study.

Number	Name	Primer（5'-3'）	Usage
1	*GAPDH*-F	TCGACAAGGCGAGCGGACAC	qRT-PCR
2	*GAPDH*-R	TGCACGATGCGTTGGAAATG	qRT-PCR
3	*ACT*-F	CATCCTTTGTATTCCCAACCGCTAT	qRT-PCR
4	*ACT*-R	CTCGTCGCCAATGAAGAAATCG	qRT-PCR
5	q*WcHSP100-1*F	AGCAGTCGGGTCATTCACTATTCC	qRT-PCR
6	q*WcHSP100-1*R	ATCTCGTCTGGTGGTGGCTGTT	qRT-PCR
7	q*WcHSP100-2*F	CTTTGTGCCCGATAATCCCTC	qRT-PCR
8	q*WcHSP100-2*R	CCATCGGAGGTAGCAGCATTT	qRT-PCR
9	q*WcHSP100-3*F	TCGACAGTTCTCAGCGGTAT	qRT-PCR
10	q*WcHSP100-3*R	CGTTATGGTGTTGTTCTAATCC	qRT-PCR
11	q*WcHSP90-1*F	CACCATAAAGGCTCTAAAGGACGAT	qRT-PCR
12	q*WcHSP90-1*R	AATGTGCCCAGGTTGGTTGTTAG	qRT-PCR
13	q*WcHSP90-2*F	ACCTCGTGATCCGCATTGTC	qRT-PCR
14	q*WcHSP90-2*R	CTGAGAGCCTCCATAAAGCC	qRT-PCR
15	q*WcHSP70-1*F	TGGCCTTTCAAGGTTGTTTCT	qRT-PCR
16	q*WcHSP70-1*R	CAAGGTATGATTCGGCGGTC	qRT-PCR
17	q*WcHSP70-2*F	TGAGCGTACCCAGTGCCGTGAC	qRT-PCR
18	q*WcHSP70-2*R	AAGGCGGCCAACGGAGTTAGTG	qRT-PCR
19	q*WcHSP70-3*F	AGACCTCCAACTTCCGCAACAC	qRT-PCR
20	q*WcHSP70-3*R	GCCAACATCTATCAGCGTAGCATT	qRT-PCR
21	q*WcHSP70-4*F	CAAGAAGGCGTTGGTAGATG	qRT-PCR
22	q*WcHSP70-4*R	CTGGGCTCACGACCGAAGAT	qRT-PCR
23	q*WcHSP60*-F	CTTTGGCGACAACCGCAAGTC	qRT-PCR
24	q*WcHSP60*-R	GTGTCCTCCTTTGTGATGGTGATG	qRT-PCR
25	q*WcsHSP-1*F	CCGTTTTACACCCTCGCCGACTTT	qRT-PCR
26	q*WcsHSP-1*R	CGCGGTTACAGCGTTTGCTTGC	qRT-PCR
27	q*WcsHSP-2*F	CCTCCTTGACGACGCCCTTAC	qRT-PCR
28	q*WcsHSP-2*R	TTGCTCTCGCCCGATACAGTG	qRT-PCR
29	q*WcsHSP-3*F	TTTCTACTCCCTCGCTGACTTCG	qRT-PCR
30	q*WcsHSP-3*R	CTGCACGTTCTCCTTGCTTATCC	qRT-PCR
31	q*WcsHSP-4*F	GGCTCTGACTGGTTGTGGGC	qRT-PCR
32	q*WcsHSP-4*R	GGATGATGACAGCAAGTTGGTGG	qRT-PCR
33	q*WcsHSP-5*F	AGGGAACCAGGCGGTAAACAT	qRT-PCR
34	q*WcsHSP-5*R	ACGGCAATCTGGTGGAACAAG	qRT-PCR
35	q*WcsHSP-6*F	GTCGCTCAGCCGCTTCCTTTAC	qRT-PCR
36	q*WcsHSP-6*R	CGTCGCAGTGACCGTGTTGG	qRT-PCR
37	qWcHSF-1F	TACGACCTGGTGTTGATGGAC	qRT-PCR
38	qWcHSF-1R	CTTGATGATCTCGTTCGGCT	qRT-PCR
39	qWcHSF-2F	GTCCACCTCGGCATCGTCCCTAT	qRT-PCR
40	qWcHSF-2R	GCCGCACAACAGGAATGGTACTCAC	qRT-PCR
41	qWcHSF-3F	TTGAAAAGCGATACAGACACCG	qRT-PCR
42	qWcHSF-3R	CGCTGAATGAGGCACAATAAGT	qRT-PCR

43	qWcHSF-4F	CACCATCCTCGGCTCGCACTTC	qRT-PCR
44	qWcHSF-4R	CGGGGCGTCCGGTTGATCTTGT	qRT-PCR
45	*WcHSP100-1*F	ATGGCTTCGAGCTTTAACTTTACCG	Cloning gene
46	*WcHSP100-1*R	TCAGTCCATTTCTTCGATCTCGATG	Cloning gene
47	*WcHSP90-1*F	ATGCGGTTCTTCCGCCCGCTGTTG	Cloning gene
48	*WcHSP90-1*R	TCACAGCTCATCATGTTTTACGGGGTT	Cloning gene
49	*WcHSP70-1*F	CTAGTCGATCTCCTCCATCGCCGACGT	Cloning gene
50	*WcHSP70-1*R	TCACAGTTCGTCATGAGAGTGAAT	Cloning gene
51	*WcHSP70-2*F	ATGGAGGACGTGTTCGACGG	Cloning gene
52	*WcHSP70-2*R	TCACCGGATACCAGCAGTGGC	Cloning gene
53	*WcHSP70-4*F	ATGGAGGGCAAGACGTCGCG	Cloning gene
54	*WcHSP70-4*R	TTAGTCCTTCTTCTCCTCCTTCTT	Cloning gene
56	m*WcHSP100-1*F	ACAGCCCAGATCTGGGTACCATGGCTTCGAGCTTTAACTTTACCG	Recombination to pET32a (+)
57	*mWcHSP100-1R*	*TGGTGCTCGAGTGCGGCCGCGTCCATTTCTTCGATCTCGATG*	Recombination to pET32a (+)
58	*mWcHSP90-1F*	*ACAGCCCAGATCTGGGTACCATGCGGTTCTTCCGCCCGCTGTTG*	Recombination to pET32a (+)
59	*mWcHSP90-1R*	*TGGTGCTCGAGTGCGGCCGCCAGCTCATCATGTTTTACGGGGTT*	Recombination to pET32a (+)
60	*mWcHSP70-1F*	*ACAGCCCAGATCTGGGTACCATGTCCCTCCCCCGTCGACCGAC*	Recombination to pET32a (+)
61	*mWcHSP70-1R*	*TGGTGCTCGAGTGCGGCCGCCAGTTCGTCATGAGAGTGAAT*	Recombination to pET32a (+)
62	*mWcHSP70-2F*	*ACAGCCCAGATCTGGGTACCATGGAGGACGTGTTCGACGG*	Recombination to pET32a (+)
63	*mWcHSP70-2R*	*TGGTGCTCGAGTGCGGCCGCCCGGATACCAGCAGTGGCTT*	Recombination to pET32a (+)
64	*mWcHSP70-4F*	*ACAGCCCAGATCTGGGTACCATGGAGGGCAAGACGTCGCG*	Recombination to pET32a (+)
65	*mWcHSP70-4R*	*TGGTGCTCGAGTGCGGCCGCGTCCTTCTTCTCCTCCTTCTT*	Recombination to pET32a (+)
